# Evaluation, management and future perspectives of anal pruritus: a narrative review

**DOI:** 10.1186/s40001-023-01018-5

**Published:** 2023-02-02

**Authors:** Matas Jakubauskas, Audrius Dulskas

**Affiliations:** 1 Department of Abdominal and General Surgery and Oncology, National Cancer Institute, Santariskiu Str. 1, 08406 Vilnius, Lithuania; 2 SMK University of Applied Social Sciences, Vilnius, Lithuania; 3 Institute of Clinical Medicine, Faculty of Medicine, Vilnius University, Vilnius, Lithuania

**Keywords:** Pruritus ani, Diagnosis, Treatment, Itch, Skin disease

## Abstract

**Purpose:**

The without a time limitation. Most recent search was performed on 1st June 2022.

**Results:**

Thorough history and physical examination are very important in view of multiple possible causes of anal pruritus. Most of the focus during examination is drawn on to the perianal region. A digital rectal examination and an anoscopy are essential. It is necessary aim of this narrative review is to overview the classification, diagnostics, possible treatment options and future perspective of anal pruritus.

**Methods:**

The search was performed by two authors (AD and MJ) independently in the following electronic databases: PubMed, EMBASE, Web of Science, Cochrane Library, CENTRAL and the Allied and Complementary Medicine Databases (AMED). Search was restricted to English language only to avoid moisture and the use of soaps in the perianal region. Furthermore, the patient should avoid certain foods and increase the intake of fiber. If the symptoms do not resolve, topical steroids, capsaicin (0.006%) and tacrolimus (0.1%) ointments may be used. For intractable cases, intradermal methylene blue injection might give a long-lasting symptom relief.

**Conclusion:**

Anal pruritus is a long-term deteriorating quality of life issue. Most of the time it is a symptom with a difficult diagnosis. Thorough history and examination should be performed for the best possible treatment.

## Introduction

Anal pruritus is defined as a condition characterized by itching around the perianal region. It affects around 1–5% of the general adult population, and it is four times more common in men than women [1]. Although, not life threatening, when long lasting this condition can greatly impair patient’s quality of life and even result in psychological issues [2, 3]. It is mainly categorized as either primary (idiopathic) or secondary, being a consequence of causal pathology. The main linking factor of the idiopathic pruritis is thought to be an increased fecal contamination of the anal region [4, 5]. Several studies showed abnormal sphincter relaxation leading to fecal soiling and perianal irritation initiating what is called an itch–scratch cycle [6–8]. In this narrative review we overview the classification, diagnostics and possible treatment options of anal pruritus.

## Materials and methods

The systematic literature search was performed in the following electronic databases: PubMed, EMBASE, Web of Science, Cochrane Library, CENTRAL and the Allied and Complementary Medicine Databases (AMED). The search consisted of terms: “anal itch”, “pruritus ani”, “anal pruritus”, “perianal sore”, “perianal burning”, “chronic itch”, “chronic pruritus”, “perianal dermatitis”.

The search was restricted to English language publications only without a time limitation. Most recent search was performed on 1st June 2022. Both authors reviewed articles independently for inclusion. Additionally, the reference lists were searched, and relevant studies were included.

### Review

#### Clinical features, diagnosis and classifications

Patients with anal pruritus present with itching, burning and soreness in the perianal region subsequently leading to scratching [6]. One of the crucial diagnostic goals is to differentiate between primary and secondary pruritus. Literature data are contradicting which of the types is more common [6, 9]. There are up to 100 conditions leading to perianal itching, which makes differential diagnostics and treatment quite challenging (most frequent can be seen in Table 1) [10–13]. In view of that, a thorough history and digital examination is of great importance.

**Table 1:** Main causes of secondary anal pruritus

Infections	Gonorrhea
	Syphilis
	Herpes simplex virus
	Human papillomavirus
	*Staphylococcus aureus*
	Beta-hemolytic streptococcus
	Erythrasma (*C. minutissimum*)
	Candidiasis
	Pediculosis
	Pinworms
	Scabies
	Human immunodeficiency virus
Anorectal disease	Hemorrhoids
	Anal fistula
	Fissures
	Rectal prolapse
	Inflammatory bowel diseases
Neoplastic lesions	Acanthosis nigricans
	Extramammary Paget’s disease
	High-grade squamous intraepithelial neoplasia
	Squamous cell carcinoma
	Melanoma
Inflammatory skin disorders	Contact dermatitis
	Atopic dermatitis
	Irritant contact dermatitis
	Psoriasis
	Urticaria
	Seborrhea
	Lichen planus
	Lichen sclerosus
Systemic diseases	Diabetes mellitus
	Renal failure
	Cholestatic liver disease
	Thyroid disorders
	Leukemia, lymphoma
	Iron deficiency anemia

The onset of the disease varies, but more often patients experience itch during the night or in the hot weather. It is necessary to inquire if the patient noticed any factors that exaggerate or alleviate the symptoms, for example, tight clothing that promotes sweating may make the itching worse [14]. Residue from detergents on clothing may also amplify the symptoms [10]. In several studies tobacco use, alcohol and several food products, such as milk, chocolate, citrus or tomatoes have been linked to idiopathic anal pruritus [9, 15]. Furthermore, patients’ hygienic practices are important, the use of soaps, perfumes and the frequency of cleansing must be documented. Inquiry about their previous patch testing and other past medical history is important as some of them could be directly linked to the development of pruritus (Table 1). Severe allergies and hereditary conditions should be noted when taking the family history. Anal pruritus in several family members is uncommon, but infectious diseases should be excluded in such context [16]. Moreover, the use of any medications, especially steroids and antibiotics, must be considered. Even the use of simple, over the counter and topical medications, has to be noted, as they may change the symptoms and appearance of the perianal region during physical examination [17].

A detailed proctological history is necessary; it should include questions about bowel movement frequency, consistency and if there were any recent changes. In cases when fecal incontinence is suspected, a specialized questionnaire, such as Wexner score, could be useful in objectifying and grading the severity of the problem [18]. The history should include whether the patient has any anorectal conditions, most importantly ones listed in Table 1; if the patient underwent any coloproctological procedures.

A thorough general physical examination may expose other sites of dermatological conditions, allergies and infections. Most of the focus during examination is drawn on the perianal region, however, the perineal and genital regions should not be forgotten as their inspection may indicate other, with pruritus-associated pathologies. In the early stages of the disease the perianal region seems normal and in more severe acute cases it presents with excoriations and mild erythema, when the disease progresses skin becomes thin, friable and lichenified [6, 19]. If macular erythema, hyperkeratosis and/or radial fissuring are present, a diagnosis of contact dermatitis is highly possible [20]. Atopic dermatitis is characterized by thickened skin and leathery patches and is usually hereditary with other site lesions. After a visual inspection, a digital rectal examination should follow. Anoscopy should be performed for all the patients with anal pruritus to identify commonly associated proctological diseases such as hemorrhoids, anal fistula or fissures as this would change the therapeutic approach later on [4, 6].

Unusual findings during digital rectal examination, sudden changes in bowel habits, long-standing inflammatory bowel disease and family history of adenomas or colorectal cancer should prompt the clinician to consider a colonoscopy [21]. Infectious etiologies are quite often diagnosed in patients with anal pruritus, thus microbiological investigation should be considered in a high-risk patient. To avoid a high rate of false negative results, correct sample collection, culture mediums and sample storage are crucial [4, 10]. Usually, blood samples are unnecessary unless the patient has systemic symptoms or treatment refractory disease. Ulcerous or persistent lesions require tissue biopsies, they should include both the lesion and normal skin. A case series study by Abu-Asi et al. suggests that all the patients should be patch tested for allergies as around 20% of them will have relevant allergens to be avoided [22].

Although not widely used in clinical practice, several additional classification systems have been proposed by various authors. It can be acute or chronic (lasting over 6 weeks) [23]. According to the condition of the perianal skin Kuehn et al. proposed a 4-stage classification [24]:

- Stage 1 (mild): No lesion is seen at inspection of anal verge, but patient finds palpation and/or anoscopy painful. Other anal lesions have been excluded.
- Stage 2 (moderate): Red dry skin only, at times weeping skin with superficial round splits and longitudinal superficial fissures.
- Stage 3 (severe): Reddened weeping skin, with superficial ulcers and excoriations disrupted by pale, whitish areas with no more hairs.
- Stage 4 (chronic): Pale, whitened, thickened, dry leathery, scaly, skin with no hairs and no superficial ulcers or excoriations (chronic condition)

A similar classification system was proposed by the Washington Hospital Center [25]:

- Stage 0—normal skin.
- Stage 1 is erythematous, inflamed skin.
- Stage 2 is lichenified skin
- Stage 3 is lichenified skin with erosions and ulcerations.

Additionally, one study developed the Anal Pruritus Life Quality Index to better quantify the impact that anal pruritus has on quality of life [26]. The questionnaire for index calculation includes 14 questions that are answered by the patient on a 10-point scale. Unfortunately, this index did not gain attention from other researchers and was never validated.

#### Treatment

Management of patients with anal pruritus may be challenging and long lasting. In cases when an exact etiology causing pruritus has been diagnosed, an intervention eliminating this factor usually is enough to resolve the symptoms [27]. For instance, the infectious pruritus causes should be treated with antiviral, antibiotic, antifungal or anthelminthic medicine, according to the detected microorganisms. Similarly, when a proctological disease such as hemorrhoids, anal fistula or fissure are suspected to be the underlying cause of pruritus, the treatment of these conditions usually, but not always, bring relief from pruritus symptoms [28].For idiopathic pruritus and some of the inflammatory dermatoses escalating treatment tactic is recommended [4, 10, 13, 27]. In such cases, patient education, patience and reassurance are the key to a successful outcome. This generic management for idiopathic pruritus ani is effective in more than 90% of patients [14].

Cleansing should be done several times per day using water or damp tissues and drying off completely afterward [29–31]. Removal of skin tags to ease the management of hygiene could be performed, however, patients should be carefully selected [4, 6, 32]. Excessive wiping and scrubbing of the perianal area should be avoided, especially with soaps, as it causes further mechanical damage and irritation. It is necessary to avoid humidity in the perianal region, so synthetic tight-fitting underwear should not be worn. Additionally, cornstarch powder or talc can be used to absorb excessive moisture [14]. The patient should be counseled to eliminate possibly irritating laundry detergents and perfumes. The patient should avoid coffee, coke, citrus, chocolate, alcohol, dairy products, tomatoes and spicy food [6, 19]. An addition of a high-fiber diet with fiber supplementation is recommended to absorb excessive moisture from the stool and reduce the incidence of occult fecal seepage. In cases when fiber only is not enough, antidiarrheal medicine such as loperamide can be prescribed [4].

Emollients and barrier creams are key to pruritus treatment and should be used after cleansing [33]. A pilot study by Tomi et al. included 28 patients to evaluate the efficacy of a film forming acrylate cream (Cavilon™ Durable Barrier Cream) for alleviating anal pruritus symptoms [26]. They used the Anal Pruritus Life Quality Index and concluded that the cream had a significant quality of life improvement. However, as the study was funded by the manufacturer of the cream, results should be critically appraised. Moreover, creams containing menthol can have an anti-pruritic effect [33, 34]. Another possible topical option is a mild steroid ointment (1% hydrocortisone). A randomized controlled cross-over trial proved its effectiveness, as after 2 weeks of treatment with a 1% hydrocortisone ointment, 68% and 75% of patients had itch reduction and quality of life improvement, respectively [35]. The use of more potent steroids or prolonged use of may eventually lead to dermal atrophy and worsen the condition [4, 10, 33]. In a randomized double-blind cross-over trial that enrolled 21 patients the use of 0.1% topical tacrolimus ointment managed to significantly decrease the itch intensity and frequency and resulted in symptom reduction in 68% of the patients 2 weeks after treatment It should be noted that the use of tacrolimus ointment did have a positive effect on the Dermatology Life Quality index, however the difference when compared to the placebo group was not significant [36]. The use of the other topical immunomodulator pimecrolimus was never investigated on idiopathic anal pruritus patients, however it is effective in treating itch caused by atopic dermatitis, although, as the literature shows, it is less effective than Tacrolimus [37, 38]. Another topical treatment option is 0.006% Capsaicin cream. After the initial burning sensation it provides desensitization and relief of the symptoms [34]. A study by Lysy et al. determined that the 0.006% concentration was effective in alleviating pruritus ani without the significant burning sensation, which is commonly caused by creams that are more concentrated. Furthermore, they showed in a randomized double-blind cross-over study that 0.006% Capsaicin cream decreased symptoms in 70% of the study cohort and prevented them from recurring [39].

Systemic treatment options have also been described in the literature [4]. Oral antihistamines (first-generation) may be beneficial to patients suffering from nocturnal scratching. Their inhibitory effect is negligible, however, they mainly act by sedating the patient [10, 33]. Antidepressants and neuroleptics are used in managing anal pruritus, however, their effectiveness was only tested for treating other locations pruritus [33].

**Fig. 1:**
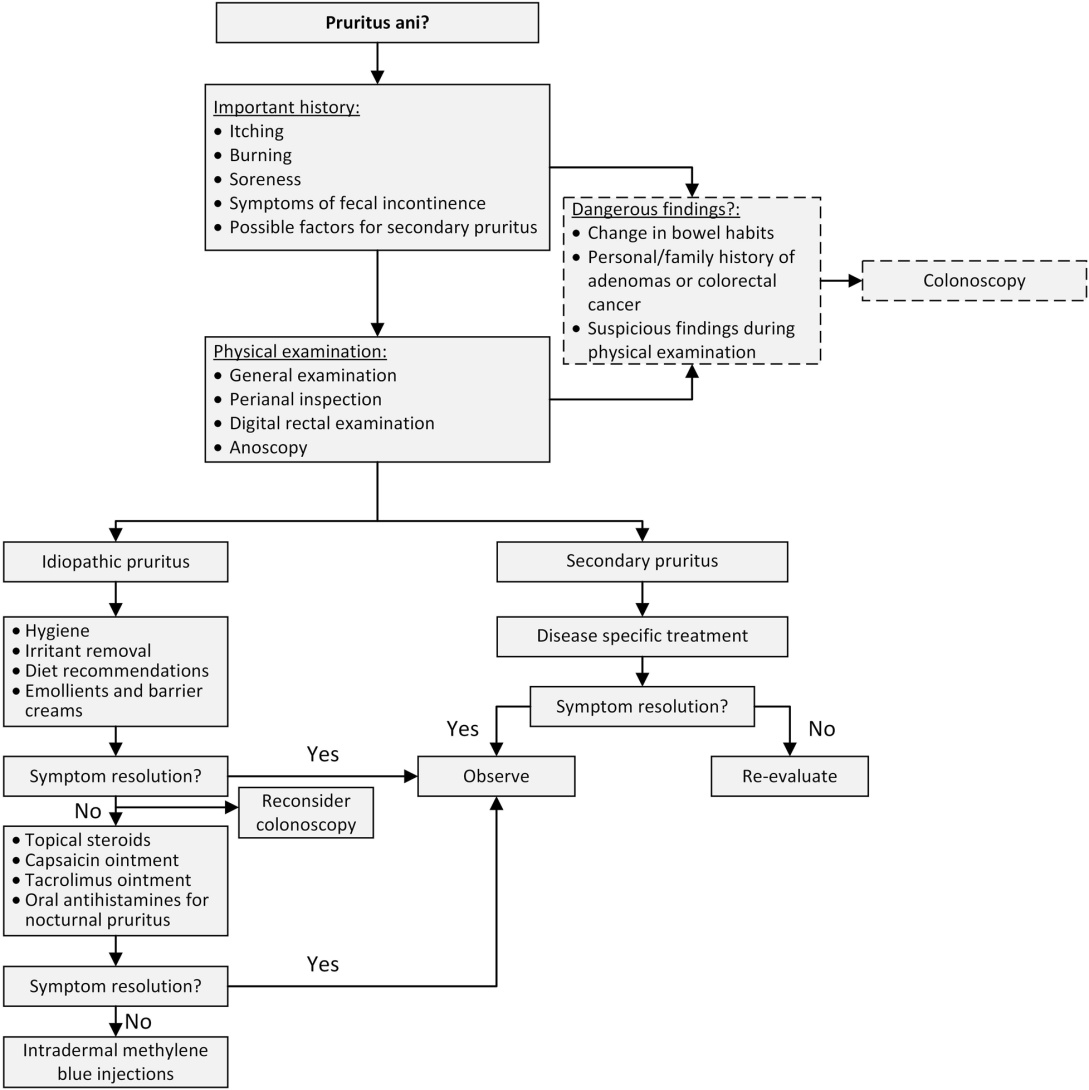
Diagnostics and treatment algorithm of pruritus ani

In long lasting intractable idiopathic pruritus cases, intradermal methylene blue injections may be used as a solution [40]. These injections act by destroying the intradermal nerve endings [41]. Eusebio et al. in their study patients under intravenous anesthesia injected up to 30 ml of 0.5% methylene blue to perianal area [41]. Up to 9.5 years of follow-up showed that 10 out of 23 patients had complete relief. Unfortunately, during the study three patients developed full thickness skin necrosis and required surgical debridement, this could be probably explained by the large injected volumes, as other studies used several times smaller and did not experience such complications [42]. Several following studies, with few technical variations, confirmed successful and long lasting results [42–44]. However, a recent literature review concluded that larger clinical studies are needed to gather stronger evidence on this treatment method [45]. In Fig. 1, we summarize the diagnostics and treatment for patients with suspected anal pruritus.

#### Future perspectives

The last few decades of extensive research into the neuroimmune itch pathways provided the foundation for novel therapeutics of chronic itch [46, 47]. Although, none of the further discussed medication have been tested on patients having anal pruritus, but they have great potential in being effective, as they mainly target different parts of itch sensation circuitry [46]. One of the most promising novel agents are monoclonal antibodies targeted against various inflammatory molecules. One of the first to get the FDA approval was dupilumab, which occupies IL-4 receptor α, thus blocking the effects of IL-4 and IL-13 [48]. It showed a broad spectrum of anti-itch action as it significantly alleviated chronic itch for patients with atopic dermatitis, chronic prurigo nodularis [49–51]. Furthermore, as the systematic review by Gael et al. show, the use of dupilumab can help 14% of patients with chronic idiopathic itch achieve full resolution and up to 89% can reach marked improvement. Lebrikizumab and tralokinumab are directed against IL-13 and act on the same pathway as dupilumab. However, differently to dupilumab, their use case is more limited as they have been mostly tested on patients with atopic dermatitis [52]. Less investigated, but also quite promising agents are nemolizumab, an IL-31 receptor α antibody, and an experimental agent vixarelimab (NCT03816891) targeting oncostatin M (OSM) receptor β block the IL-31/OSM pathway and thus exert anti-pruritic effect [53]. These two antibodies were mainly tested on inflammatory dermatoses, thus their effects on idiopathic anal pruritus remains questionable [54, 55].

Another breakthrough is being seen in the use of JAK/STAT signaling axis inhibitors. They act by disturbing immune cell cytokine release and have been used in managing several other autoimmune diseases and even COVID-19 infection [46, 56, 57]. The main adverse event is increased severe infection rate [58]. Mostly investigated agents for pruritus are baricitinib, abrocitinib, upadacitinib and tofacitinib, however, currently they have been tested mainly on patients with atopic dermatitis [59–62]. Additionally topical forms of JAK inhibitors could be beneficial to patients with anal pruritus. A phase 2 randomized trial by Kim et al. investigated the use of ruxolitinib on atopic dermatitis patients. They concluded that significant effects of ruxolitinib begin to appear after 2 weeks of use and the maximum efficacy is achieved by week 4. Moreover, in a phase 3 trial, Nakagawa et al. showed that delgocitinib ointment can significantly improve patients modified Eczema Area and Severity Index scores, reaching the maximum efficacy by week 4. Importantly both topical agents had limited adverse events [63, 64].

The research into the disruption of opioid system showed that µ receptor antagonists and κ receptor agonists may be used in controlling chronic itch [65]. The µ receptor antagonists, namely naloxone and naltrexone have been developed to treat opioid induced adverse effects. However, as Lee et al. showed, naltrexone can be also advantageous in managing chronic pruritus of various origin [66]. In their study, naltrexone greatly reduced pruritus intensity to 13 of the 18 (72.2%) included patients that had pruritus of different etiologies. The κ receptor agonists (nalfurafine and difelikefalin) are relatively new agents and have been mainly tested in reducing uremic pruritus on patients undergoing hemodialysis [67–69]. Additionally, nalbuphine acting as both a κ agonists and µ antagonists, was effective in treating uremic pruritus and prurigo nodularis [70, 71].

Although initial results of these novel treatment agents are promising, further research is warranted to determine their safety and effectiveness for treating idiopathic anal pruritus.

## Conclusion

Anal pruritus is a chronic condition which can affect quality of life. It can be difficult to establish the primary cause as pruritus often is secondary to underlying condition. Thorough history and examination should be performed for the best possible treatment. Treatment consists of a stepwise approach focusing mainly on patient education and the use of topical agents.

## Data Availability

Not applicable.
